# Generation and characterization of a double-knockout Arabidopsis thaliana line lacking expression of AOX1a and VTC2

**DOI:** 10.18699/vjgb-26-27

**Published:** 2026-04

**Authors:** E.V. Garmash, E.S. Belykh, K.V. Yadrikhinskiy, R.V. Malyshev, I.O. Velegzhaninov

**Affiliations:** Institute of Biology of Komi Science Centre of the Ural Branch of the Russian Academy of Sciences, Syktyvkar, Russia; Institute of Biology of Komi Science Centre of the Ural Branch of the Russian Academy of Sciences, Syktyvkar, Russia; Institute of Biology of Komi Science Centre of the Ural Branch of the Russian Academy of Sciences, Syktyvkar, Russia; Institute of Biology of Komi Science Centre of the Ural Branch of the Russian Academy of Sciences, Syktyvkar, Russia; Institute of Biology of Komi Science Centre of the Ural Branch of the Russian Academy of Sciences, Syktyvkar, Russia

**Keywords:** Arabidopsis thaliana, alternative oxidase, ascorbate, GDP-L-galactose phosphorylase, AOX1a and VTC2 knockout lines, crossing, genotyping, double mutants, lethal mutation, Arabidopsis thaliana, альтернативная оксидаза, аскорбат, ГДФ-L-галактозофосфорилаза, линии с нокаутом АОХ1а и VTC2, скрещивание, генотипирование, двойная нокаутная линия, летальная мутация

## Abstract

In higher plants, the L-galactose pathway is the main pathway for the biosynthesis of vitamin C (ascorbate, Asc), the final step of which is connected with the functioning of the mitochondrial electron transport chain (ETC). In addition to the main cytochrome pathway, plant ETC includes an alternative pathway (AP) via alternative terminal oxidase (AOX). The engagement of AOX promotes Asc synthesis, and it is hypothesized that AOX suppression under conditions of Asc deficiency may reduce plant viability. The aim of this work was to examine the consequences of simultaneously knocking out two genes in Arabidopsis thaliana: AOX1a, the most stress-inducible AOX gene, and VTC2, encoding a key enzyme of the L-galactose pathway of Asc synthesis. Two lines of A. thaliana with T-DNA insertions in the target genes were crossed to generate hybrid lines. Seed characteristics of the first (F1) and second (F2) generations were analyzed. F1 seeds were larger than those of parent lines, possibly due to heterosis. In the F2 generation, self-pollination of F1 plants resulted in seeds with significant size variation, including a group of small seeds with degenerative morphological abnormalities. Most of small seeds failed to germinate or died at the seedling stage. PCR genotyping of these seeds revealed the absence of native AOX1a and VTC2 indicating a lethal mutation caused by simultaneous knockout of both genes. One likely genetic cause is the interaction of mutations in non-allelic genes. At the physiological level, irreversible respiratory damage may occur, possibly including the impact of a cryptic mutation in the vtc2 line. Further studies are necessary to confirm these hypotheses. In general, the results obtained indicate the vital co-functioning of the AP and the L-galactose pathway of Asc biosynthesis and may be useful for the development of genetically engineered techniques for the control of vitamin C synthesis in plants

## Abstract (ru)

У высших растений L-галактозный путь является основным путем биосинтеза витамина С (аскорбата, Аск), последний этап которого связан с функционированием электрон-транспортной цепи митохондрий. Помимо основного цитохромного пути, электрон-транспортная цепь растений содержит альтернативный путь через альтернативную терминальную оксидазу (АОХ). Вовлечение АОХ способствует синтезу Аск, поэтому предполагается, что подавление альтернативного пути в условиях дефицита Аск может привести к снижению жизнеспособности растений. В связи с этим была поставлена цель – рассмотреть последствия нокаутирования в растениях Arabidopsis thaliana одновременно двух генов: АОХ1а, наиболее стресс-индуцибельного гена АОХ, и VTC2, гена ключевого фермента L-галактозного пути синтеза Аск. Для этого проведено скрещивание двух линий A. thaliana с Т-ДНК инсерцией в целевых генах и получены гибридные линии. Изучены характеристики семян первого (F1) и второго (F2) поколений. Семена F1 отличались более крупными размерами по сравнению с родительскими линиями, что могло быть следствием гетерозиса. В поколении F2 в результате самоопыления растений F1 сформировались семена, значительно варьирующие по размерам, в том числе группа мелких семян, имеющих дегенеративные морфологические отклонения. Большинство мелких семян не прорастало или погибало на стадии появления проростка. Генотипирование этих семян с помощью ПЦР установило отсутствие нормальных копий АОХ1а и VTC2 в геноме, что указывает на появление летальной мутации при одновременном нокауте обоих генов. Обсуждаются причины летального исхода двойного нокаута. Одной из генетических причин, по-видимому, стало взаимодействие мутаций (неаллельных генов). На физиологическом уровне, возможно, возникли необратимые нарушения дыхания, в том числе из-за влияния в линии vtc2 криптической мутации. Для подтверждения данных гипотез требуются дальнейшие исследования. В целом полученные результаты свидетельствуют о жизненно важном совместном функционировании альтернативного и L-галактозного путей биосинтеза Аск и могут быть полезны для развития генно-инженерных приемов управления синтезом витамина С в растениях.

## Introduction

**Fig. 1.**
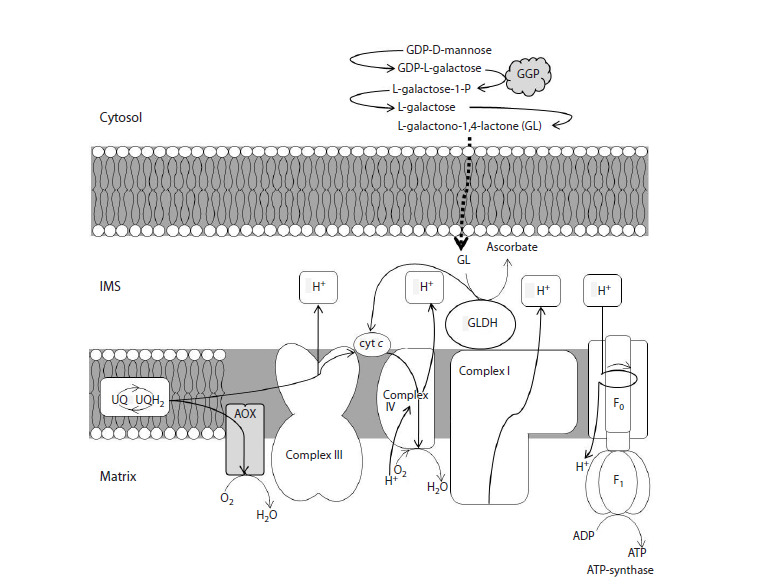
Schematic representation of the relationship between ascorbate synthesis and the electron transport chain (ETC) of plant mitochondria The ETC components in the inner mitochondrial membrane are shown: from the ubiquinone pool (UQ/UQH2) electrons are transferred via the alternative pathway (AP) catalyzed by alternative oxidase (AOX) and via the cytochrome pathway through complex III, cytochrome c, and complex IV. GGP denotes GDP-L-galactose phosphorylase. L-galactono-1,4-lactone (GL), synthesized through the L-galactose pathway and transported from the cytosol into the mitochondrial intermembrane space (IMS), is oxidized to ascorbate by L-galactono-1,4-lactone dehydrogenase (GLDG), which transfers electrons to cytochrome c. Electron transport along the ETC is coupled with proton pumping into the intermembrane space and the “return” of protons into the matrix via ATP synthase, driving ATP production

Ascorbate (vitamin C) is an important multifunctional antioxidant that participates in stress resistance and redox signaling (Smirnoff, 2018; Foyer et al., 2020; Matos et al., 2022, and others). Plants replenish their ascorbate pool through several mechanisms, including biosynthetic processes and regeneration via the ascorbate-glutathione cycle. Among these pathways, the L-galactose pathway is the dominant one (Dowdle et al., 2007; Wheeler et al., 2015). In the L-galactose pathway, GDP-D-mannose is converted into GDP-L-galactose, L-galactose- 1-phosphate, L-galactose, L-galactono-1,4-lactone, and finally into L-ascorbate (Asc) (Fig. 1). A key step in this pathway is the reaction catalyzed by GDP-L-galactose phosphorylase (GGP) (Yoshimura et al., 2014; Matos et al., 2022). The GGP enzyme is encoded by the paralogous genes VTC2 and VTC5 (VITAMIN C), which demonstrate light-dependent expression patterns (Yoshimura et al., 2014; Smirnoff, 2018). Among these, VTC2 plays the primary role in Asc biosynthesis. Knockout of VTC2 (unlike its paralog VTC5) results in a significant decrease (up to 80 % or more) in Asc levels and plant growth (Dowdle et al., 2007; Lim et al., 2016).

The final stage of the L-galactose pathway is associated with the mitochondrial electron transport chain (ETC), where L-galactono-1,4-lactone dehydrogenase (GLDG) oxidizes L-galactono-1,4-lactone to Asc by transferring electrons between complexes III and IV via the labile electron carrier cytochrome c (Fig. 1). Unlike mammalian ETC, plants, in addition to the main energy-conserving cytochrome pathway (CP), possess an alternative pathway for electron transport (AP) through an alternative terminal cyanide-resistant oxidase (AOX) (Garmash, 2022) (Fig. 1). The AP bypasses two sites of proton translocation, complexes III and IV, and therefore represents a non-energy-conserving branch of electron transport (Vanlerberghe et al., 2020). The main function of the AP is to maintain redox balance during mitochondrial electron transport and to reduce reactive oxygen species (ROS) production. This impacts the metabolism of not only mitochondria but also cells and plants as a whole (Del-Saz et al., 2017; Vanlerberghe et al., 2020; Garmash, 2022). Moreover, the AP promotes Asc synthesis by accepting electrons from ubiquinone and maintaining the cytochrome c pool in a more oxidized state (Bartoli et al., 2006; Matos et al., 2022).

Studies of mutant lines with VTC2 suppression reveal a number of defects and metabolic disturbances, especially under stressful conditions (Lim et al., 2016; Matos et al., 2022). Ascorbate deficiency and the stunted phenotype observed in these mutant plants likely result from long-term impact of the mutation on respiratory activity, mitochondrial ETC function, and, in particular, on electron transfer via AOX (Matos et al., 2022).

Nuclear genome of A. thaliana includes five genes of АОХ, with the highest expression in response to various types of stress attributed to the gene АОХ1а (Del-Saz et al., 2017; Garmash, 2022). A. thaliana plants overexpressing АОХ1а demonstrated increased Asc synthesis under stress conditions (high light) (Garmash et al., 2022; Sweetman et al., 2022), whereas mutant lines with АОХ1а suppression showed decreased Asc levels (Vishwakarma et al., 2015; Garmash et al., 2022). Additionally, A. thaliana plants of the vtc2 line exhibited АОХ activation compared with wild-type plants, and treatment of mutant plants with a specific inhibitor of the AP resulted in a significant (up to 40 %) decrease in ATP concentration (Garmash et al., 2024). Other authors (Talla et al., 2011) studied responses of A. thaliana plants with knockout of the GDP-mannose phosphorylase gene catalyzing GDP-D-mannose synthesis (vtc1 line) on mitochondrial and chloroplast ETC inhibition, demonstrating mutual complementation between Asc and АОХ, preventing excess accumulation of ROS and protecting photosynthesis from photooxidation. These data confirm the participation of АОХ in maintaining Asc synthesis and optimizing energy balance and indicate that suppression of the AP under conditions of Asc deficiency can result in decreased plant viability. Therefore, we hypothesized that AOX1a and VTC2 play a key role in providing energy and vital functions of the plant organism.

The aim of this paper was to study the consequences of simultaneous knockout of two genes, АОХ1а and VTC2, in A. thaliana plants. To this end, mutant lines were crossed, and the characteristics of seeds from the first (F1) and second (F2) generations were analyzed. Double knockouts were confirmed by T-DNA insertion localization using primers specific to the target genes

## Materials and methods

**Fig. 2.**
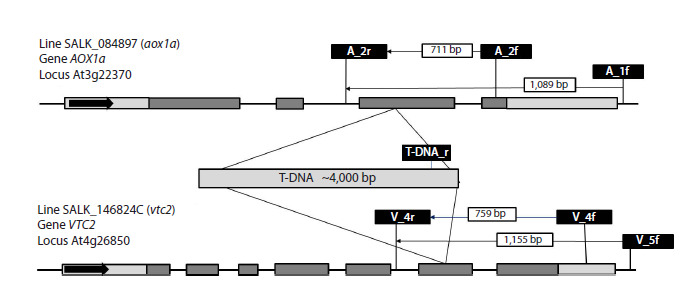
Scheme of primer locations, PCR product lengths, and T-DNA insertion sites in the АОХ1а gene from line SALK_084897 (аох1а) and VTC2 from line SALK_146824C (vtc2). Primer pairs V_4f-V_4r and V_5f-V_4r, as well as A_2f-A_2r and A_1f-A_2r, allow identification of the wild type VTC2 or АОХ1а genes, respectively. Primer pairs V_4f-T-DNA_r and V_5f-T-DNA_r, as well as A_2f-T-DNA_r and A_1f-T-DNA_r, allow identification of the corresponding mutant genes.

Mutant lines with T-DNA insertion into АОХ1а – SALK_084897 and into VTC2 – SALK_146824C were used for hybridization (Fig. 2).

Seeds of mutant lines were obtained from the Arabidopsis Biological Resources Centre (ABRC, Ohio University, USA). The line with knockout of АОХ1а (аох1а) contains a T-DNA insertion in one copy of the АОХ1а gene, making the line aox1a heterozygous. This line also has an insertion in the EFS gene, which encodes the flowering inhibitor histone-lysine N-methyltransferase. The knockout line for VTC2 (vtc2) has insertions in both copies of the gene and is homozygous. The wild-type line Columbia-0 (Col-0) was used to control for relative transcript content. Seeds of Col-0 were obtained from the Nottingham Arabidopsis Stock Centre (NASC, UK) and kindly provided by O.I. Grabelnykh and V.I. Tarasenko (SIPPB SB RAS).

Seeds were planted, stratified, and germinated in 200 cm3 pots containing a mixture of perlite, vermiculite, and soil in a 1 : 1 : 2 ratio. Seedlings were grown under controlled conditions at 22 °C with a light intensity of 90 μmol quanta/ (m2 · s) and a 16-hour photoperiod until the budding phase (stage 5.1; Boyes et al., 2000). Lighting was provided by luminescent lamps (TL-D 30W and TL-D 30WAquarelle, Philips, Netherlands).

Hybridization was performed by transferring pollen from the line SALK_146824C (vtc2) onto the line SALK_084897 (aox1a) under microscopic control. For this purpose, unblown buds (one day before blooming) on the maternal plant were carefully opened and dissected to remove all stamens with pollen with tweezers. Pollen from the paternal plant was transferred onto the stigma of the maternal plant’s pistil. The fertilized flower was marked, and fertilization was repeated for three consecutive days. All surrounding flowers were regularly removed during the budding phase. The plant was isolated with a transparent plastic cylinder until seed maturation.

Seed size from the F1 and F2 generations was measured (n = 111 and 496 for F1 and F2, respectively). The length (major semi-axis of the ellipse, a, mm) and width (minor semi-axis of the ellipse, b, mm) of the seeds were measured under a microscope. The ellipse area (S, mm2) was calculated as S = a·b·π. F2 seeds were divided according to size into three groups: large, medium, and small (n = 49, 365, and 82 for each group, respectively).

To check for embryo presence, some seeds from each group, swollen after soaking in water, were dissected under a microscope.

To analyze the relative levels of gene transcripts, genotyping and qPCR were performed on plant samples at growth stage 1.14 (Boyes et al., 2000) and on seeds.

To genotype the parental lines (аох1а, vtc2) and crossbred lines, DNA from the corresponding samples was used along with primers (Fig. 2, Table 1). DNA was extracted from 50–100 mg of air-dried leaves or dry seeds using the Sorb- GMO-B kit (Sintol, Russia) according to the manufacturer’s instructions. The PCR reaction mixture contained 4 μL of Screen Mix (Evrogen, Russia), 4 μL of each corresponding forward and reverse primer (0.3 μM) (Sintol, Russia), 7 μL of ddH2O (PanEco, Russia), and 1 μL of DNA. Amplification consisted of a preliminary denaturation for 5 minutes at 95 °C, followed by 35 cycles of denaturation (15 s) at 95 °C, primer annealing (30 s) at 55 °C, and elongation (60 s) at 72 °C, with a final elongation for 2 minutes at 72 °C. PCR products were analyzed by electrophoresis on a 1 % agarose gel in Tris-Acetate buffer. DNA fragments were stained with Safe Green Stain (APGENA, Russia) and visualized with blue light using the SuperRay Maxi system (APGENA, Russia). Fragment lengths were determined using a 100+ bp DNA ladder (100–1,500 bp) (Evrogen, Russia).

**Table 1.**
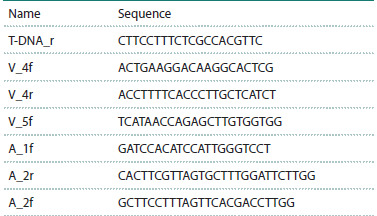
Primers for genotyping and checking for T-DNA insertions in the VTC2 gene (V) of line SALK_146824C (vtc2), and the AOX1a gene (A) of line SALK_084897 (aox1a)

Relative levels of gene transcripts were analyzed by qPCR using the CFX96 system (Bio-Rad, USA). Rosette leaves were frozen in liquid nitrogen and stored at –80 °C prior to analysis. RNA was extracted using the HiPure Plant RNA Kit (Magen, China) according to the manufacturer’s instructions. RNA concentration was measured using Qubit (Thermo Fisher Scientific, USA) with the Qubit™ RNA HS Assay Kit (Thermo Fisher Scientific, USA). For cDNA synthesis, the MMLV RT kit with oligo(dT) primers (Evrogen, Russia) was used. qPCR reactions were performed with “qPCRmix-HS SYBR” (Evrogen, Russia) and the corresponding primers (Table 2). Target gene expression was normalized to the geometric mean of the AT2G28390 and AT4G34270 reference genes (Czechowski et al., 2005). Relative transcript levels were calculated using the 2−ΔΔCT method (Livak, Schmittgen, 2001). Analyses were performed on three biological replicates, including leaves from one crossbred plant or 3–5 individual plants of the original lines, with two technical replicates for each sample.

**Table 2.**
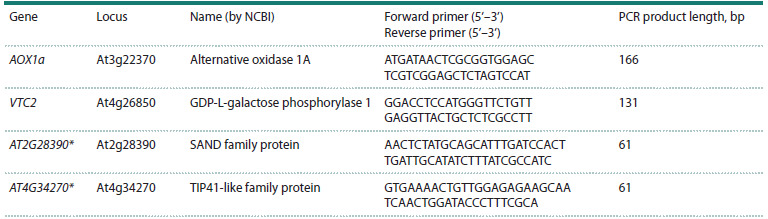
The list of primers for qPCR in A. thaliana Note. Primers were designed using Primer-BLAST (Ye et al., 2012). * – reference genes (from Czechowski et al., 2005).

## Results

Genes AOX1a (At3g22370), VTC2 (At4g26850), and EFS (At1g77300) are located on chromosomes 3, 4, and 1 of A. thaliana, respectively. The offspring of heterozygotes obtained by crossing lines SALK_084897 and SALK_146824C, according to unlinked inheritance and Mendel’s third law, allow obtaining all possible combinations of variants in the F1 generation, and homozygous offspring for both target mutations in the F2 generation.

**Fig. 3.**
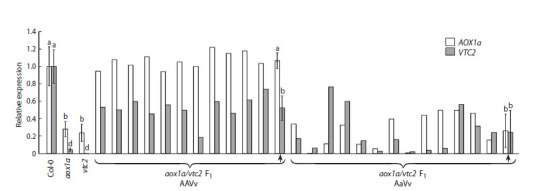
Relative levels of АОХ1а and VTC2 transcripts in leaves of A. thaliana plants from the wild-type line (Col-0), parental lines (аох1а and vtc2), and 24F1 hybrids (aox1a/vtc2). Arrows indicate mean values and their standard errors for the presumed genotypes (AAVv and AaVv). Statistical significance of differences in gene expression levels is indicated by different letters (ANOVA, Duncan’s test, p ≤ 0.05).

**Fig. 4.**
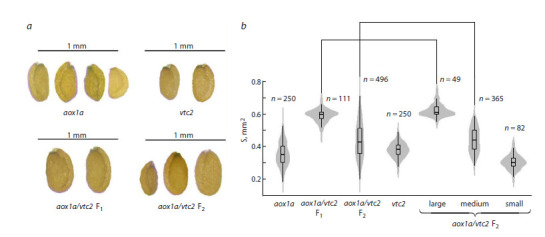
Appearance (a) and seed size (S) (b) of parental and crossbred (aox1a/vtc2) A. thaliana lines from the F1 and F2 generations. Panel (b) shows seed size distribution presented as violin plots combined with boxplots, including arithmetic mean values and standard errors. n indicates sample size. Differences between paired parameters not marked with a square bracket are statistically significant at p < 0.01 (Dunn’s test with the Bonferroni correction).

**Fig. 5.**
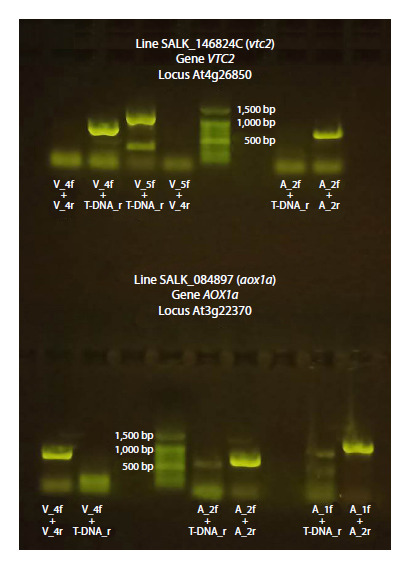
Electrophoregram of DNA amplicons from A. thaliana plants of the vtc2 and aox1a lines, demonstrating the presence of T-DNA insertions in the VTC2 or АОХ1а genes correspondently. Primer descriptions are provided in Table 1.

Relative transcript levels of АОХ1а obtained by qPCR in the аох1а line were significantly lower than in the Col-0 line, and the level of VTC2 mRNA in the vtc2 line was nearly undetectable (Fig. 3). Seed size of the aox1a line also varied notably compared to the vtc2 line (Fig. 4). These data indirectly confirm the heterozygosity of aox1a and the homozygosity of vtc2 mutant alleles, which was further verified by the presence of T-DNA insertions in the corresponding lines (Fig. 5). Bands corresponding to PCR products with primer pairs V_4f-V_4r and V_5f-V_4r, specific to wild-type genes, were absent, while bands with primer pairs V_4f-T-DNA_r and V_5f-T-DNA_r, confirming T-DNA insertion, were present in the electrophoregram of the vtc2 line. The T-DNA insertion was located precisely at position 13,499,579 in the sixth exon of the gene At4g26850 in the vtc2 line (Belykh et al., 2024). The presence of a band with primer pair A_2f-A_2r and the absence of one with A_2f-T-DNA_r indicated the wild-type status of the AOX1a gene

The аох1а line, in contrast, is characterized by a band corresponding to the PCR product of the V_4f-V_4r primer pair and the absence of a band corresponding to V_4f-T-DNA_r, confirming the presence of a wild-type copy of the VTC2 gene (Fig. 5). Bands corresponding to both primer pairs for the АОХ1а gene (A_2f-A_2r and A_2f-T-DNA_r; A_1f-A_2r and A_1f-T-DNA_r) indicate the coexistence of a wild-type copy together with a T-DNA insertion-disrupted copy, characteristic of a heterozygous line.

To predict segregation in the F1 generation, a scheme based on a Punnett square was drawn up to determine possible genotype variants of the offspring based on parental genotypes and their gametes, calculated by dividing the number of cells of a given genotype by the total number of cells. According to the scheme, segregation by genotype was 1 : 1 : 1 : 1 (four phenotypes in equal proportions) (Table 3). In other words, 25 % of F1 seeds belonged to the mutant line AaVvEE, deficient in VTC2 and АОХ1а; 25 % had genotype AaVvEe, deficient in all genes (АОХ1а, VTC2, EFS); the remaining seeds were represented by genotypes deficient in VTC2 or in VTC2 and EFS.

**Table 3.**
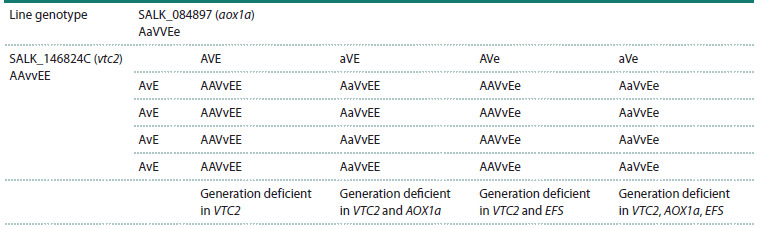
Punnett square for the F1 generation from crossing lines SALK_146824C (vtc2) and SALK_084897 (аох1а) Note. A – wild-type (as in Col-0) AOX1a gene; a – insertion in АОХ1а; V – wild-type (as in Col-0) VTC2 gene; v – insertion in VTC2; E – wild-type (as in Col-0) EFS gene; e – insertion in EFS.

F1 seeds obtained from the cross were significantly larger than those from the parental lines (Fig. 4). This likely results from heterosis, manifested as increased hybrid viability due to the inheritance of diverse alleles from genetically distinct parents.

Plants grown from 24 F1 seeds exhibited decreased VTC2 transcript levels (Fig. 3). More than half (54 %) also showed decreased АОХ1а transcript levels. The genome of the remaining 46 % of plants presumably contained both copies of АОХ1а, as in wild-type plants. Thus, the hybridization corresponded well with the predicted segregation

**Fig. 6.**
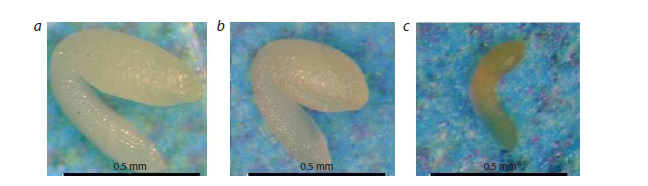
Embryos dissected from large (a), medium (b) and small (c) seeds of A. thaliana crossbreed lines aox1a/vtc2 of the F2 generation

Following self-pollination, the F1 plants produced fruits containing F2 seeds. These seeds varied considerably in size (Fig. 4) and were classified into three significantly different groups. Notably, the largest seed group did not differ in size from the F1 seeds, suggesting genotypes of either AAVv or AaVv, as in the F1 generation. The smallest seeds exhibited degenerative morphological abnormalities; dissection revealed undeveloped embryos (Fig. 6). Most small seeds failed to germinate or died at the seedling stage.

**Fig. 7.**
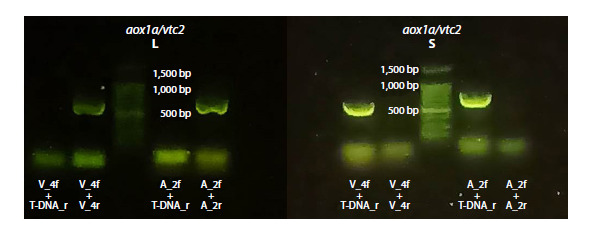
Electrophoregram of DNA amplicons from large (L) and small (S) seeds of the F2 generation from the A. thaliana crossbreed aox1a/vtc2 lines. The presence of bands corresponding to primer pairs V_4f-V_4r and A_2f-A_2r, characteristic of the wild-type variants of the VTC2 and АОХ1а genes, respectively, is evident in large seeds and absent in small seeds. Primer descriptions are provided in Table 1.

Genotyping of large seeds from the F2 generation revealed the presence of wild-type copies of the АОХ1а and VTC2 genes in their genomes (Fig. 7). In contrast, bands corresponding to wild-type genes were absent in the electrophoregram of DNA extracted from small seeds. This finding confirms that the genotype of small seeds corresponds to the double knockout mutant aox1a/vtc2.

## Discussion

Studies of vitamin C-deficient lines, primarily vtc2, have demonstrated the important role of the L-galactose pathway of vitamin C biosynthesis, and in particularly that of the key enzyme GGP, in replenishing the main pool of this metabolite in plants. However, it is believed that the reduced growth and viability of mutants are associated more with metabolic disturbances and an imbalance in the respiratory ETC than with decreased Asc levels alone (Lim et al., 2016; Matos et al., 2022).

It has been shown that these abnormalities may be linked to a cryptic mutation in the mutant line (Lim et al., 2016). However, the same authors demonstrated that the growth defects of vtc2 mutants segregate independently of the mutation in the VTC2 gene, indicating that the potential cryptic mutation is located on a different chromosome. This allows the analysis of the relationship between the phenotype of the offspring and the status of the VTC2 gene.

The causes of metabolic and growth disturbances of the vtc2 line have been identified in several studies using mutant combinations with this line. The growth-reduced phenotype of vtc2 has been described in combination with mutations causing chronic photooxidative stress (Müller-Moulé et al., 2004), knockout of chloroplast ascorbate peroxidase (Giacomelli et al., 2007), and suppression of the autoimmune response (Zhu et al., 2013). A double vtc2-1/abi4 mutant, generated by crossing the A. thaliana vtc2-1 line (a loss-of-function mutant with significantly decreased VTC2 transcription) with an abscisic acid-insensitive mutant line (abi4), exhibited growth and morphology similar to those of wild-type plants (Kerchev et al., 2011). This suggests that abscisic acid contributes to the stunted phenotype of vtc2-1. However, all described double knockout mutants retained the ability to grow. The only lethal phenotype was observed in the double knockout of both GGP genes (VTC2 and VTC5) (Dowdle et al., 2007).

Previously, vitamin C-deficient mutants, vtc1 (Conklin et al., 1996) and vtc2 (Jander et al., 2002), were initially selected for their sensitivity to ozone. Ozone sensitivity likely results from imbalances in respiratory pathways and increa- sed ROS generation due to low Asc synthesis. Inhibition of the cytochrome pathway under elevated ozone levels occurred simultaneously with a sharp increase in AOX activity (Ederli et al., 2006; Pasqualini et al., 2007). These findings support the hypothesis that enhanced AOX activity in the vtc2 line protects against of oxidative stress and possible plant death.

This hypothesis was confirmed in our study, where the F2 generation was nonviable when both AOX1a and VTC2 were knocked out simultaneously. It is possible that the lethal mutation resulted from the interaction of mutations in non-allelic genes, including a cryptic mutation. However, the role of the interaction between the AOX1a and VTC2 mutations – rather than between AOX1a and the cryptic mutation – is supported by the observation that all F2 seeds that failed to germinate carried a mutation in VTC2, which appears to segregate independently from the cryptic mutation (Lim et al., 2016). To clarify this issue, more detailed molecular genetic studies are needed, including genomic DNA sequencing and RNA analysis to identify mutations affecting pre-mRNA splicing.

## Conclusion

As a result of crossbreeding of the SALK_084897 and SALK_146824C lines, seeds of varying sizes were obtained in the F2 generation. The smallest seed group was characterized as aberrant, displaying degenerative morphological deviations and an inability to germinate. Genotyping of these seeds revealed the absence of both AOX1a and VTC2, indicating a lethal mutation arising from the simultaneous double knockout of these genes. This finding highlights the essential joint functioning of two systems: the alternative pathway through AOX and the L-galactose pathway of Asc biosynthesis linked to the mitochondrial ETC. Further investigation into the interrelationship and coordination between the AP and the L-galactose Asc synthesis pathway could focus on producing double mutants with defects in genes involved in ascorbate synthesis, other respiratory electron transport components, and participants in hormonal and redox signaling. Such research may aid in the development of genetic engineering strategies to enhance ascorbate synthesis, thereby improving stress tolerance in significant food-value crops.

## Conflict of interest

The authors declare no conflict of interest.
